# The contributions of social comparison to social network site addiction

**DOI:** 10.1371/journal.pone.0257795

**Published:** 2021-10-28

**Authors:** Hyunji Kim, Richard Schlicht, Marlit Schardt, Arnd Florack

**Affiliations:** Faculty of Psychology, University of Vienna, Vienna, Austria; University of Zurich, SWITZERLAND

## Abstract

Excessive use of social network sites (SNSs) can often lead to negative consequences of frequent upward social comparisons despite having the social network platform to present users in a favorable light. However, the existing literature gives little evidence to social comparison related antecedents and consequents of uncontrollable use of SNSs. The present study aimed to investigate the contributions of social comparison to SNS addiction. In Study 1, using a convenient sample in Austria (*n* = 103), we showed that the tendency to engage in social comparisons of ability (but not of opinion) predicted self-reported SNS addiction over and above the feelings of relative deprivation on social support and status. SNS addiction mediated the relations between social comparison of ability and stress, but not self-esteem. In Study 2, using a broad sample of participants in Austria (*n* = 500), we replicated the findings observed in Study 1 and showed that contrastive upward social comparison emotions (i.e., envy, depression) mediated the relation between SNS addiction and lower self-esteem whereas the contrastive downward social comparison emotion (i.e., contentment) mediated the relation between SNS addiction and higher self-esteem. Our findings suggest that SNS addiction closely relates to psychological constructs relevant to social comparison, mediates the link between social comparison of ability and detrimental consequences (i.e., stress, well-being) and demonstrate how social comparison emotions relate to both positive and negative associations between SNS addiction and self-esteem.

## Introduction

Recent technological developments transformed the way people interact with each other from face-to-face to mobile-device-dependent interactions. Many people have utilized social network sites (SNSs) such as *Facebook*, *Twitter*, or *Instagram* allegedly for means to stay in touch with friends or to build new social connections and this trend has highly contributed to the boom of online social interactions in the recent decade [1, 2].

While SNSs have enabled us to make new social connections beyond physical restrictions [3, 4], empirical findings have shown that excessive use of SNSs is associated with detrimental psychological effects. Despite some positive effects such as enabling effective information sharing and strengthening group cohesion [5], extensive evidence suggests that excessive SNS activities are associated with negative consequences such as increased anxiety [6], lower self-esteem [6–10], higher negative affect [11], lower positive affect [12, 13], depression [7, 14–17] burnout [18], and decreased mental and physical health (e.g., [19, 20]), to the point that can induce malfunctioning social behavior which often requires clinical treatments (e.g., [14, 21]). Given the negative impacts on general health in the society, investigating social and individual indicators that contribute to developing problematic SNS use is highly warranted.

Interestingly, recent empirical evidence has suggested that some of the negative psychological consequences of SNS activities might be attributable to engaging in frequent adverse social comparisons on social network platforms [11–14]. For instance, the tendency to engage in social comparison was associated with poorer well-being led by passive browsing [22, 23]. Engaging in upward social comparisons on SNS platforms led to lower self-esteem [24]. Frequent online adverse social comparisons were associated with worsened health for individuals with depressive symptoms and low self-esteem [25, 26] and also associated with compulsive buying in women [27]. Overall, accumulative empirical evidence has indicated that the tendency to engage in online social comparisons might partly underlie the detrimental impact of SNS use (for a review, see [28]).

SNS platforms certainly provide social environments whereby individuals can make social comparisons easily and more quickly because the comparison information is immediately available and apparent in SNS posts [18, 24, 29]. Comparison related indicators such as “Likes” are also readily available in most SNS platforms [30]. Although engaging in social comparisons is crucial for self-evaluation and self-improvement purposes [31], making social comparisons has more complex consequences that can lead to positive and negative psychological effects: Upward social comparisons can lead to self-enhancement and boost self-motivation (assimilating the self with the comparison target) but also induce envy or resentment (contrasting the self from the comparison target) whereas downward comparisons can lead to satisfaction and relief (contrasting the self from the comparison target) but also induce fear of becoming the comparing target (assimilating the self with the comparison target; e.g., [32, 33]). However, SNS users are more likely to engage in upward than in downward comparisons because of the nature of SNS environment being a platform to advertise positive life styles, events, success stories, and flattering or entertaining posts [34], partly to make positive impressions about the self to peers [35, 36], colleagues, friends, and other socially connected acquaintances [37, 38]. Due to such omnipresent ideals in SNSs, engaging in social comparisons might evoke stronger negative than positive consequences for individuals.

So far, the existing literature on SNS social comparison and its psychological effects mainly speaks to the normal range of SNS behavior whereby reducing the frequency of SNS activities could be an effective cure for lessening the negative consequences (e.g., [39]). Although drawing a clear border between normal and abnormal SNS use is difficult [40], a challenge arises when the SNS usage behavior falls into the problematic range whereby SNS users essentially suffer from being addicted to engage in SNS activities. Severe SNS addiction shares many characteristics with other behavioral addictions such as pathological gambling [41, 42], which renders the potential remedy to be more complex and painstaking. However, to what extent psychological antecedents that might explain SNS addiction is limited. In particular, the link between psychological constructs surrounding social comparison (e.g., relative deprivation, self-esteem, social comparison elicited emotions) and SNS addiction has not been investigated to date. Given that SNS platforms provide ample opportunities and easy access to engaging in social comparisons, and given the complex effect (positive and negative consequences) of social comparisons that are fundamental to securing the self-concept in social life, what motivates the SNS users to continuously use SNS platforms despite negative impacts (i.e., stress, self-esteem) might be partly attributable to the tendency to engage in social comparisons.

In fact, the reported negative association between self-esteem and SNS addiction alone might not fully explain why individuals who report low self-esteem continuously go back to using SNS platforms despite the negative consequences. Based on the function of social comparison, possibly an occasional positive experience after engaging in SNS social comparisons such as the feeling of inspiration or contentment might partly account for this behavior. Although social comparison is ubiquitous, a large individual difference exists (e.g., [43, 44]). And the impact of individual social comparison might be better captured by gauging social comparison relevant emotions that are the cascade of positive and negative psychological consequences of engaging in social comparison activities. We speculated that gauging social comparison relevant emotions (e.g., inspiration, envy, anger, depression, sadness, contentment; [45]) might clarify the dynamics of SNS addiction and self-esteem because engaging in social comparison itself is not necessarily harmful but rather, it depends on what emotions were elicited as a consequence of making such comparisons. Further, investigating this question might help explain why problematic SNS users continue to misuse SNSs despite the negative psychological costs.

In the present study, we investigated the raised questions directly. More specifically, we examined the tendencies to engage in social comparisons of ability and opinion, perceived social and material relative deprivation as antecedents (Study 1) and social comparison emotions as consequences (Study 2) of SNS use, and tested how these variables affect stress, well-being, and self-esteem.

To date, a few research lines that identified psychological antecedents of SNS addiction have suggested some predispositional individual characteristics such as extraversion, neuroticism, narcissism, need for social contact or individual feelings for “fear of missing out” [46–48]. People with depression [49], poor foresight and impulsivity [50] were more vulnerable to develop SNS addiction. Related to the tendency to engage in social comparisons, people who reported low self-esteem were more likely to show problematic use of SNSs [51–54]. In order to boost self-esteem, individuals often engage in social comparisons in a strategic way by comparing the self with others who are worse off, to feel better about themselves (contrastive downward comparison; [55]) or by comparing themselves with others who are similar but slightly better off, to view the self as a person who could achieve the same (assimilative upward comparison; [56]). However, SNS users who aim to boost their self-esteem might actively engage in SNS social comparisons while constantly failing to do so, because other users portraying themselves in a rose-tinted image.

As a result, the more individuals engage in SNS activities, the more they might feel envious or eventually depressed about themselves. Recent empirical evidence supports this view by showing that upward social comparisons on SNS platforms indeed can lead to lower self-esteem [24], especially those who report a higher tendency to engage in social comparisons [57, 58]. Based on previous evidence, we investigated whether the tendency to engage in SNS social comparisons would be associated with lower self-esteem and whether this relation is mediated by SNS addiction.

Another limitation of the existing literature is that most empirical research on social comparison and SNS behavior have rarely made a distinction between the tendencies to engage in social comparison of ability and of opinion (c.f. [59]). The ability and opinion comparisons differ in what purpose they serve. While opinion comparison serves to identify similarities and differences in values, attitudes and beliefs with others, ability comparison serves to identify one’s relative standing on various social outcomes [31, 60–62]. Due to its competitive nature and self-evaluative function, ability comparison is highly likely to cause negative psychological consequences when engaging in social comparisons (e.g., [63, 64]). Therefore, we separated the measure for tendency to engage in social comparisons by the contents of comparisons (ability or opinion). We expected that the tendency to make social comparisons of one’s ability but not one’s opinion would predict uncontrollable SNS behavior because opinion-based social comparisons are not grounded in one’s social outcomes, and thus contribute little to self-evaluation [62]. SNS addictive behaviors might arise once SNSs are used as a tool to engage in easy social comparisons on how well one is performing in various types of social outcomes such as social relationships, jobs, material wealth, lifestyle and social status because the more social comparisons one makes the more self-evaluation materials one might attain. This social comparison motive (i.e., tendency to engage in social comparisons) might accelerate SNS use to the extent that it becomes an uncontrollable habit. Undeniably, SNS platforms are useful for presenting apparent social outcomes (e.g., material possession, social status), which creates a suitable environment for making ability comparisons. It is therefore important to distinguish opinion and ability comparisons when investigating its detrimental consequences in the field.

To test the predictive power of the tendencies to engage in social comparisons of ability and opinion, and its surrounding constructs on SNS addiction, we first focused on two potential predictors; one is the tendency to engage in social comparisons and another is the general tendency to feel or perceive oneself as relatively deprived on social support and status when comparing oneself with similar others. The feeling of relative deprivation is induced when individuals compare themselves with similar others on various social outcomes and feel that one is deprived of something they deserve to have [65]. The feeling of relative deprivation on social outcomes has been shown to explain a higher tendency to make risky choices and engage in gambling behavior in order to cope with such negative feelings [66, 67]. Likewise, as SNSs provide a convenient platform whereby people can freely connect with others and make new connections, people who tend to feel higher relative deprivation on social relationships, material wealth and social status might use SNSs excessively in an effort to eliminate negative feelings.

To summarize, we first examined the predictive power of social comparison relevant constructs namely, social comparisons of ability and opinion, personal relative deprivation of social status and relationships on SNS addiction. Secondly, we aimed to test whether SNS addiction mediates the relation between the high level of tendency to engage in social comparison of ability and the negative consequences on mental health (i.e., stress, low self-esteem). We list our hypotheses as follows.

H1. Social comparison of ability but not opinion predicts SNS addiction.
H2. Social comparison of ability predicts SNS addiction over and above other variables.
H3a and H3b. SNS addiction mediates the relation between social comparison of ability and stress (H3a), and self-esteem (H3b).

In Study 1, we tested our hypotheses using a convenient sample (n = 103). In Study 2, we replicated our findings using a sample from a market research panel with broader demographic characteristics (n = 500) while additionally testing the mediation effect on well-being and underlying mechanisms of the relation between SNS addiction and self-esteem via social comparison elicited emotions.

## Study 1

### Materials and methods

#### Participants

Our sample size was calculated in advance to achieve over 80% power with a small to medium effect size (*f* ^2^ = .15). Participants in Austria were recruited freely for a voluntary participation of a 10-minute survey. A hundred and eleven participants (33 males, *M*_age_ = 27.62, *SD*_age_ = 7.68) were recruited via email and Facebook. Eight participants failed the attention check and were therefore excluded (e.g., “Please indicate the mid-point of the scale”).

#### Procedure and measures

Participants completed the measures listed below. The order of presenting the measures below was random across participants. All translated items are included in the supporting information.

*Personal Relative Deprivation Scale (PRDS)*. We used back-translation methods [68] with two translators both fluent in English and German to translate Callan et al.’s [66] 5-item Personal Relative Deprivation Scale and measured individual relative deprivation on material wealth and social status. Example items are “I feel deprived when I think about what I have compared to what other people like me have”, “I feel resentful when I see how prosperous other people like me seem to be”. (see S1 Table) Participants indicated how strongly they agreed with each item given a 6-point scale (1 = *strongly disagree* to 6 = *strongly agree*). Higher scores implied higher relative deprivation on material wealth and status.

*Social Personal Relative Deprivation Scale (SPRDS)*. To measure participants’ individual relative deprivation on social support (i.e., close friendship), we adapted the back-translated version of the PRDS items and replaced “material wealth” or “social status” with “close friendships” and measured the feeling of social personal relative deprivation. Participants indicated to what extent they agreed with each item given a 6-point scale (1 = *strongly disagree*, 6 = *strongly agree*). Example items are “I feel deprived when I think about how many close relationships I have compared to what other people like me have”, “I feel resentful when I see how many close relationships other people like me seem to have”. (see S4 Table). Higher values indicated higher deprivation on close social relationships.

*Iowa Netherlands Comparison Orientation Measure (INCOM)*. We used a previously validated German translated version of INCOM to measure participants tendency to engage in social comparisons [69]. 6 items measured participants’ tendency to engage in social comparison of ability (e.g., “I often compare myself with others with respect to what I have accomplished in life”) and 5 items measured tendency to engage in social comparison of opinion (e.g., “I often try to find out what others think who face similar problems as I face”). Higher scores indicated stronger tendency to engage in social comparisons.

*SNS addiction*. To assess behavioral tendencies to engage in uncontrollable use of SNSs, we back-translated the Bergen Social Media Addiction Scale (BSMAS; [70]) with two translators both fluent in English and German (see S7 Table). The 6-item BSMAS measured the extent to which people thought that they had failed to control their excessive engagement in SNS activities. Example items were “How often during the last year have you felt an urge to use social media more and more?”, “How often during the last year have you tried to cut down on the use of social media without success?”. Participants indicated how often they experienced each item given a 5-point scale (1 = *never*, 5 = *very often*). Higher scores indicated higher tendency to engage in excessive and unwanted SNS activities.

*Self-esteem*. A single item measure, “I have high self-esteem” [71] was back-translated and used to measure self-esteem using a 5-point scale (1 = *not very true of me*, 5 = *very true of me*; see S1 Scale).

*Perceived stress*. A single item measure, “How much stress (e.g., because of hassles, demands) were you under recently?” [72], was back-translated and used to measure felt stress using a 5-point scale (1 = *felt very slightly or not at all*, 5 = *felt very much*; see S2 Scale).

*Income and education*. As previous studies examining subjective social status have often controlled for objective social status (income and education; [66, 67]), we have included measures for income and education to control for participants’ objective social status. Participants reported their annual household income before tax given 10 categories (1 = *less than €10*,*000*, 2 = *€10*,*000 – €14*,*999*, 3 = *€15*,*000 – €19*,*999*, …, 10 = *€50*,*000 or more*) and indicated their highest level of educational attainment given four categories (1 = *did not finish high school*, 2 = *high school graduation*, 3 = *college graduation*, 4 = *postgraduate degree*). Income responses were converted into estimates of absolute income applying Parker and Fenwick’s [73] median-based estimator.

### Results

First, following the procedure of Kim et al.’s Korean translated version of PRDS [63], we performed exploratory factor analyses for German translated versions of measures of perceived relative deprivation as regards material wealth (PRDS) and social support (SPRDS) to confirm a simple structure. Exploratory factor analysis revelated that German PRDS items loaded on two factors, showing items 1, 3, and 5 loading onto one factor (eigenvalue = 2.54, 50.73% variance explained), two reversed items (items 2 and 4) loading onto a separate factor (eigenvalue = 1.27, 25.34% variance explained; see S2 Table). Following Wong, Rindfleisch, and Burroughs’ [74] recommendations, we used a 3-item PRD scale (items 1, 3, and 5) in our further analyses to measure perceived relative deprivation of material wealth. The German translated version of SPRDS showed a one factor structure, eigenvalue = 2.75, 55.01% variance explained (see S5 Table), and therefore we used a 5-item SPRDS to assess the perceived relative deprivation according to the social support in our further analyses.

Descriptive statistics and correlations among measures are shown in Table 1. All translated measures showed acceptable internal validities (all alphas > .65; see Table 1). Our results revealed that the tendency to engage in social comparisons of ability was significantly positively correlated with SNS addiction (1–1.99 = 53.4%, 2–2.99 = 34.9%; 3–3.99 = 8.8%; 4–4.99 = 2.9%), *r*(101) = .49, *p* < .001, perceived relative deprivation of material wealth, *r*(101) = .39, *p* < .001, tendency to engage in social comparisons of opinion, *r*(101) = .58, *p* < .001, and negatively correlated with self-esteem *r*(101) = -.21, *p* = .036, but did not correlate with perceived relative deprivation of social relationship, *r*(101) = .14, *p* = .166, nor with perceived stress, *r*(101) = .13, *p* = .182. SNS addiction was highly positively correlated with social comparison tendencies (ability: *r*(101) = .49, *p* < .001, opinion: *r*(101) = .28, *p* = .004), and perceived relative deprivation of material wealth, *r*(101) = .33, *p* = .001, and perceived stress, *r*(101) = .27, *p* = .007, but did not correlate with perceived relative deprivation of social relationship, *r*(101) = .04, *p* = .707 nor with self-esteem, *r*(101) = -.17, *p* = .08. Although self-esteem did not show a significant correlational result in our sample, the coefficient value we observed was comparable to the overall coefficient value (*r* = -.18) calculated in a meta-analysis of the relation between self-esteem and problematic SNS use [54]. Thus, it seems that our sample might have lacked power to detect a small effect of a such correlation. Overall, our correlational results indicated that tendency to engage in social comparisons of ability might be highly relevant to social comparison motives, perceived deprivation of material wealth, and stress, but not to perceived relative deprivation of social support.

**Table 1:** Descriptive statistics and intercorrelations for measures in Study 1.

Measures	*M* (*SD*)	1.	2.	3.	4.	5.	6.	7.	8.	9.
1. SC-Ability	2.83 (.82)	(.83)								
2. SC-Opinion	3.65 (.67)	.58**	(.65)							
3. RD Social Support	2.14 (.85)	.14	.10	(.78)						
4. RD Material Wealth	2.27 (.80)	.39**	.15	.21*	(.81)					
5. Self-Esteem	3.66 (.88)	-.21*	-.08	-.33**	-.26*	-				
6. Stress	3.63 (.95)	.13	.05	-.04	.05	-.10	-			
7. SNS Addiction	1.95 (.75)	.49**	.28**	.04	.33**	-.17	.27**	(.80)		
8. Income (€)	17.2k (12.8k)	-.17	-.07	-.25**	-.18	.10	.17	-.09	-	
9. Education	3.50 (.78)	.10	.06	-.17	-.27**	.01	-.002	-.03	.11	-

*Note*. SC = Social Comparison; RD = Relative Deprivation. SNS = Social Network Site. When applicable, alpha reliability are presented in parentheses along the diagonal.

* *p* < .05.

** *p* < .01.

Next, we conducted a linear multiple regression analysis to examine the predictive power of the tendency to engage in social comparisons and feelings of relative deprivation on SNS addiction. The regression analysis revealed that our predictors explained 26% variances of SNS addiction (*R*^2^ = .26), *F*(4, 98) = 8.75, *p* < .001. As reported in Table 2, the tendency to engage in social comparisons of ability (*β* = .42, *p* < .001) accounted for SNS addiction over and above perceived relative deprivation of material wealth (*β* = .17 *p* = .077), and social support (*β* = -.06, *p* = .513) and tendencies to engage in social comparisons of opinion (*β* = .01 *p* = .909; see Table 2). This result was consistent after controlling for income and education. Our data suggested that the accountability of tendencies to engage in social comparisons of ability on SNS addiction was not confounded by social comparisons of opinion, and social and material relative deprivation at the individual level. In other words, SNS addiction seems to be mainly driven by urges to engage in social comparisons of ability but neither by the urges to engage in social comparisons of opinion nor by the effort to cope with perceived relative deprivation.

**Table 2:** Multiple regression analysis in Study 1.

	SNS addiction			
Predictors	*b* (*se*)	95% CI for *b*	*β*	*sr* ^2^
SC-ability	.39 (0.11)	.14, .56	.42**	.32
SC-opinion	.01 (0.12)	-.21, .26	.01	.01
RD Social Support	-.05 (0.08)	-.21, .11	-.06	-.06
RD Material Wealth	.14 (0.08)	-.02, .28	.17	.16

*Note*. SC = Social Comparison; RD = Relative Deprivation. SNS = Social Network Site. *sr*^2^ = semi-partial correlation squared.

* *p* < .05.

** *p* < .01.

**Fig 1:**
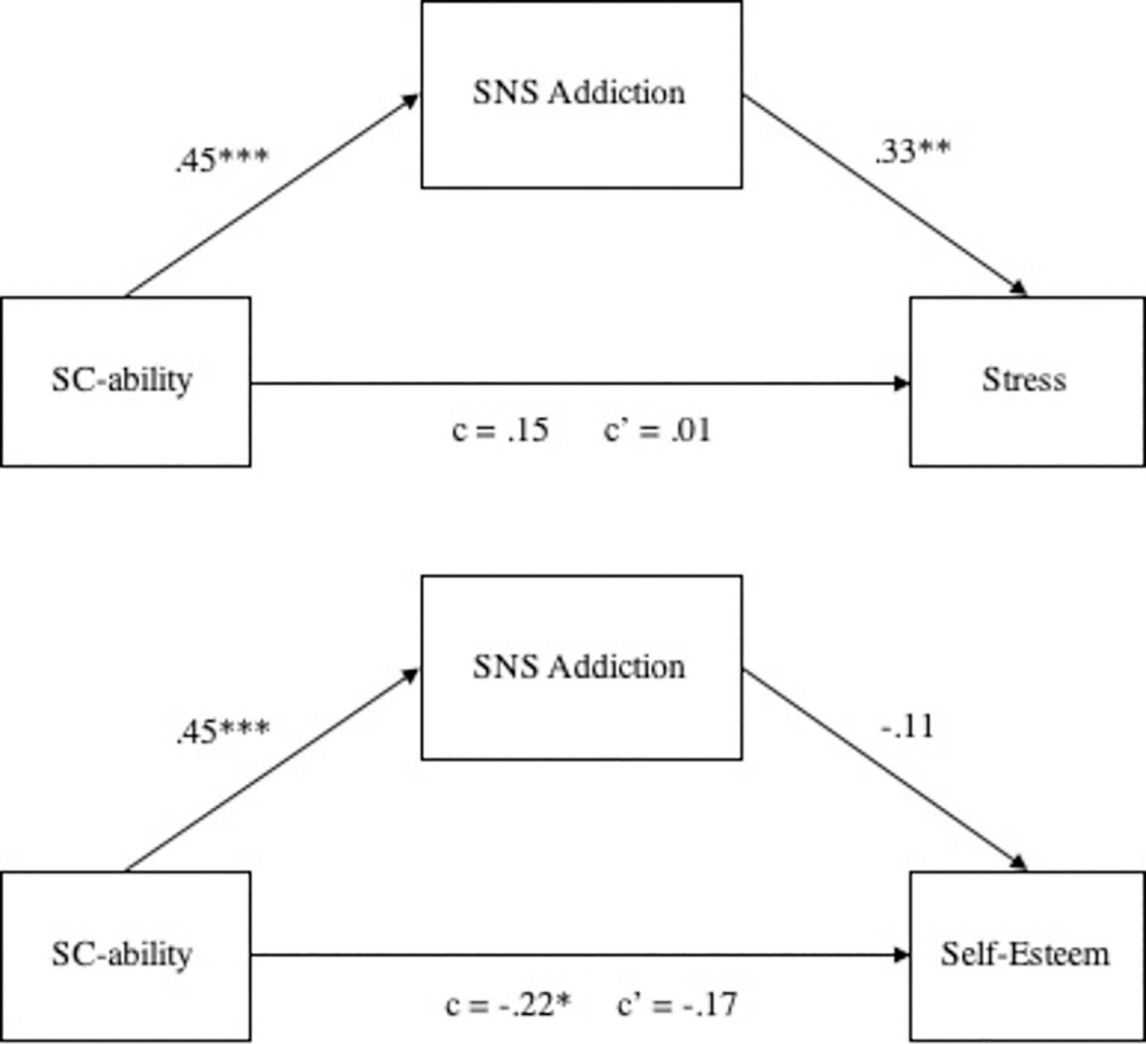
The mediating role of SNS addiction on the relations between social comparison of ability and stress, and self-esteem.

Lastly, we ran two mediation analyses testing the mediating role of SNS addiction on the relations between social comparison of ability and stress, and self-esteem (Fig 1). Bootstrapped mediation analyses with 10,000 samples (PROCESS, [75]) revealed that SNS addiction fully mediate the relation between social comparison of ability and stress (95% BCa CI of -.04 and .29; total effect = .15; indirect effect = .15, *SE* = .07), confirming our Hypothesis 3a. However, SNS addiction did not significantly mediate the relation between social comparison of ability and self-esteem (95% BCa CI of -.18 and .05; total effect = -.22; indirect effect = -.05, *SE* = .06), showing that our Hypothesis 3b was not confirmed.

### Discussion

Our results showed that a higher tendency to engage in social comparison of ability uniquely predicted SNS addiction among other social comparison constructs tested in Study 1. Based on our finding, SNS addiction might be driven by the motivation to use SNSs as a tool for making quick social comparisons on one’s social outcomes but not by the desire for attaining social resources that one feels deprived of. SNS addiction indeed mediated the relation between social comparison of ability and stress. However, our data was based on a convenient sample that limits the conclusion and generalization of the present findings. To draw a stronger conclusion and test for further hypotheses, we conducted Study 2 while including measures of well-being and social comparison elicited emotions as additional dependent variables.

A surprising finding of Study 1 was the non-significant mediation model between the social comparison tendency of ability and self-esteem through SNS addiction. This finding was unexpected given the previous evidence on the negative association between SNS addiction and self-esteem and its close association with social comparison tendency. Nevertheless, although a recent meta-analysis showed that self-esteem is moderately negatively correlated with problematic SNS use (*r* = -.18; [54]), the existing evidence on the relation between self-esteem and SNS use is rather mixed. While some researchers found a negative association between self-esteem and SNS use [76, 77] others have found a positive association [78, 79]. We speculated that the reason for the non-significant mediation might be the positive and negative associations between SNS use and self-esteem cancelling each other out in one model. To test this possibility, we investigated a potentially mixed mechanism through the lens of SNS social comparisons in the next study. In other words, we investigated whether positive and negative social comparison emotions resulting from engaging in social comparison activities differentially mediate the relation between SNS addiction and self-esteem.

More specifically, to unravel the underlying mechanism among SNS addiction, social comparison, and self-esteem, we investigated whether the contrastive upward social comparison emotions (envy, anger, and depression) would mediate the relation between SNS addiction and lower self-esteem. Likewise, we also investigated whether positive social comparison emotions such as contentment and inspiration would mediate the relation between SNS addiction and higher self-esteem. Despite the feelings of envy and depression after making upward social comparisons on SNS platforms, individuals might also experience some doses of positive emotions after making a contrastive downward or assimilative upward comparison that might boost their self-esteem, reinforcing them to return to SNS sites. As this potential connection has not been tested empirically, we investigated this link in the next study.

## Study 2

In Study 2, we hypothesized that SNS addiction will mediate the relation between social comparison of ability and stress (replicating H3a), well-being (H3c), but not self-esteem (replicating the non-confirmative H3b). Lastly, we expected to observe the mediating effect of contrastive upward social comparison emotions (i.e., envy, anger, depression) in the relation between SNS addiction and lower self-esteem (H4a), and the mediating effect of positive social comparison emotions (i.e. inspiration, contentment) in the relation between SNS addiction and higher self-esteem (H4b). While replicating our findings observed in Study 1, our additional hypotheses were tested as below.

H3c. SNS addiction mediates the relation between social comparison of ability and well-being.
H4a. Contrastive upward social comparison emotions (i.e., envy, anger, depression) mediate the relation between higher SNS addiction and lower self-esteem.
H4b. Contrastive downward social comparison emotions (i.e., contentment) mediate the relation between higher SNS addiction and higher self-esteem.

In Study 2, we also included a measure to assess participants’ SNS behavior such as frequency and duration of SNS activities, types of SNS sites being used, types of device used for engaging in SNS activities.

### Materials and methods

#### Participants

Five hundred participants (243 males, *M*_age_ = 43.46, *SD*_age_ = 14.56), recruited via a professional survey platform as a sample with broader demographic characteristics in Austria (respondi.com), entered the analyses after excluding sixty participants who failed one of more of the three attention check items included in the survey (e.g., “Please indicate the mid-point of the scale”) and one participant who reported the age below eighteen. Participants took approximately 10 minutes to complete the survey.

#### Procedure and measures

Participants gave an online informed consent by clicking a button to continue and complete the survey. At the beginning of the survey, participants reported whether they were an SNS user or not. Only those who reported that they were SNS users proceeded to the survey and completed the measures per Study 1, and additionally, participants reported their SNS behavior and emotions they felt after engaging in social comparisons in an SNS environment, as described below.

*SNS behavior*. Participants reported their SNS behavior by completing a back-translated German version of the SNSUN scale (part 1 from Social Networking Sites Usage and Needs Scale; SNSUN, [80]). In this scale, participants indicated what devices they use for SNS activities, what type of SNS platforms they use and how frequent each SNS platform is being used (see Tables 4 and 5).

*Well-being*. Participants filled out a German translated version of SPANE (The Scale of Positive and Negative Experience; [81]) to report how frequent they experienced positive and negative emotions in the last 4 weeks. Participant indicated how often they experienced each emotion given a 5-point scale (1 = *very rarely or never*, 5 = *very often or always*). Following the recommendation for assessing well-being as balance by Diener et al. [82], we subtracted the mean values of the negative emotions (i.e., feeling negative, bad, uncomfortable, sad, fearful, and angry) from the mean values of the positive emotions (i.e., feeling positive, good, comfortable, happy, joyful, and content) to form a balance score as an indicator for subjective well-being. In this composite well-being score, higher values indicated higher level of well-being.

*SNS social comparison emotions*. To gauge emotions that arise after engaging in social comparisons on SNS platforms, we adapted a measure used by Park and Baek [83] for gauging social comparison relevant emotions on Facebook. We modified “Facebook” to “SNS platforms” and the participants read the German translated version of the item, “While using SNS platforms, how often did you feel the following emotions when you compared yourself with the networked others on SNSs?” Participant indicated their answer on a 5-point scale (1 = *never*, 5 = *always*). We selected 6 different types of emotions people feel after making upward or downward social comparisons (upward and assimilative social comparison emotions = admiration/inspiration; upward and contrastive social comparison emotions = envy, anger, depressed; downward assimilative social comparison emotions = sadness; downward contrastive social comparison emotions = content/satisfied) based on the social comparison emotion categorized by Smith [84].

### Results

Similar to Study 1, due to the German translated versions of PRDS to measure perceived relative deprivation of material wealth and SPRDS to measure perceived relative deprivation of social support both showing a two-factor structure, we removed the reverse items and used a 3-item PRDS and a 3-item SPRDS for data analyses (see S3 and S6 Tables for EFA reports).

The demographic information and participants’ SNS usage behavior are shown in Tables 3–5. The descriptive statistics and correlations among measures are shown in Table 6. All translated measures showed good internal validities (all alphas > .73). Replicating the results in Study 1, our data revealed that SNS addiction (1–1.99 = 59.6%, 2–2.99 = 32%; 3–3.99 = 7.8%; 4–4.99 = 0.6%) was highly positively correlated with social comparison tendencies (ability: *r*(498) = .43, opinion: *r*(498) = .24), and perceived relative deprivation of material wealth, *r*(498) = .35, perceived stress, *r*(498) = .20 (all *p*s < .001), and self-esteem *r*(498) = -.15, *p* = .001, but also positively correlated with perceived relative deprivation of social support this time, *r*(498) = .33, *p* < .001. Overall, our correlational results in Study 2 confirmed the overall relations between SNS addiction and all social comparison related measures, indicating that the tendency to engage in social comparisons, the perception of relative deprivation of material wealth and social support are highly associated with SNS addiction.

**Table 3:** Demographics of the sample in Study 2.

No.	Category	Value	Frequency	Percentage
**1.**	Gender	Male	243	48.6
		Female	256	51.2
		Not specified	1	0.2
**2.**	Age	18–20	17	3.4
		21–30	98	19.6
		31–40	115	23
		41–50	107	21.4
		51–60	95	19
		61–70	45	9
		71–80	23	4.6
**3.**	Educational attainment	Did not finish high school	23	4.6
		High school graduation	186	37.2
		College graduation	162	32.4
		Postgraduate degree	129	25.8
**4.**	Household income	Less than 10.000€	23	4.6
		10.000€ - 14.999€	43	8.6
		15.000–19.999€	42	8.4
		20.000–24.999€	59	11.8
		25.000–29.999€	43	8.6
		30.000–34.999€	45	9.0
		35.000–39.999€	32	6.4
		40.000–44.999€	38	7.6
		45.000–49.999€	28	5.6
		50.000€ or more	59	11.8
		Not specified	88	17.6

**Table 4:** SNSs usage pattern reported using the SNSUN scale in Study 2.

No	Category	Value	Frequency	Percentage
**1.**	SNSs use	Often	344	68.8
		Occasionally	138	27.6
		Rarely	18	3.6
**2.**	Preferred device	Desktop computer	52	10.4
		Laptop	60	12.0
		Mobile Phone	359	71.8
		Tablet	27	5.4
		Other	2	0.4
**3.**	Number of SNS used actively	One	80	16.0
		Two	173	34.6
		Three	142	28.4
		Four	65	13.0
		Five or more	40	8.0
**4.**	Checking SNSs account per day	1–2 times per day	112	22.4
		3–4 times per day	115	23.0
		5–6 times per day	85	17.0
		7–8 times per day	51	10.2
		9+ times per day	89	17.8
		On every notification beep	48	9.6
**5.**	Time spent on SNSs per day	1–2 hours	359	71.8
		3–4 hours	113	22.6
		5–6 hours	24	4.8
		7–8 hours	3	0.6
		9 hours and more	1	0.2
**6.**	Increase in SNSs use	During the day	139	27.8
		In the evening	295	59.0
		At night	16	3.2
		On weekends	31	6.2
		Other	19	3.8
**7.**	Duration of using SNSs	Less than one year ago	8	1.6
		1–2 years ago	19	3.8
		3–4 years ago	40	8.0
		5–6 years ago	92	18.4
		7–8 years ago	78	15.6
		9–10 years ago	95	19.0
		More than 10 years ago	168	33.6

**Table 5:** SNS platforms and the frequency of using each platform reported in Study 2.

SNS Platform	Every Day	3 to 5 times a week	Occasionally	Rarely	Never
**Facebook**	61.4%	15.6%	9.4%	6.4%	7.4%
**Facebook Messenger**	11.6%	14.6%	32.6%	23.8%	17.4%
**Twitter**	5.2%	4.8%	7.8%	16.4%	65.8%
**WhatsApp**	82.2%	9.2%	3.2%	0.8%	4.6%
**Myspace**	0.2%	0.4%	1.0%	6.4%	92.0%
**Instagram**	30.8%	10.4%	13.0%	10.0%	35.8%
**YouTube**	28.4%	30.2%	28.0%	7.4%	6.0%
**Snapchat**	8.8%	3.8%	5.0%	7.8%	74.6%
**Google+**	12.0%	7.0%	10.0%	10.2%	60.8%
**LinkedIn**	2.2%	4.8%	8.8%	11.8%	72.4%
**TikTok**	3.6%	3.4%	4.8%	8.2%	80.0%
**Signal**	1.4%	1.2%	3.4%	6.0%	88.0%
**Other**	1.8%	2.2%	1.8%	1.2%	46.2%

**Table 6:** Descriptive statistics and intercorrelations for measures in Study 2.

Measures	*M* (*SD*)	1.	2.	3.	4.	5.	6.	7.	8.	9.	10.	11.	12.	13.	14.	15.	16.
1. SC-ability	2.25 (.86)	(.85)															
2. SC-opinion	3.13 (.79)	.47***	(.73)														
3. RD Social Support	2.02 (1.09)	.46***	.14**	(.80)													
4. RD Material Wealth	2.17 (1.12)	.51***	.20***	.53***	(.85)												
5. Self-Esteem	3.58 (.96)	-.31***	-.07	-.28**	-.36***	-											
6. Stress	3.13 (1.07)	.12**	.15**	.16**	.25***	-.14**	-										
7. SNS addiction	1.86 (.67)	.43***	.24***	.33***	.35***	-.15**	.20***	(.80)									
8. Income (€)	34.03k (17.07k)	.01	.02	-.05	-.14**	.14**	.03	-.08	-								
9. Education	2.79 (.88)	.03	.10*	.05	.01	.01	.13**	.08	.13**	-							
10. Well-Being	1.32 (1.44)	-.28***	-.09*	-.38***	-.47***	.41***	-.39***	-.33***	.13**	-.07	-						
11. SC-Inspiration	2.76 (1.02)	.28***	.26***	.15**	.12**	-.08	.13**	.38***	-.02	.07	-.05	-					
12. SC-Envy	1.83 (.94)	.44***	.26***	.30***	.41***	-.18***	.11*	.40***	.05	.07	-.29***	.36***	-				
13. SC-Anger	2.60 (1.01)	.19***	.13**	.12**	.18**	-.12**	.24***	.20***	.03	.07	-.29***	.24***	.26***	-			
14. SC-Sadness	2.17 (.90)	.28***	.15**	.24***	.22***	-.19**	.12**	.35***	.03	-.08	-.26***	.37***	.28***	.44***	-		
15. SC-Depression	1.83 (.90)	.39***	.25***	.32***	.38***	-.25***	.23***	.49***	-.05	.03	-.38***	.31***	.49***	.39***	.50***	-	
16. SC-contentment	2.95 (.93)	.12**	.13**	-.03	-.06	.10*	.02	.22***	.07	-.04	.23***	.40***	.11*	.10*	.12**	.08	-

*Note*. SC = Social Comparison; RD = Relative Deprivation; SNS = Social Network Site.

* *p* < .05.

** *p* < .01

*** *p <* .001.

To replicate the regression model tested in Study 1, we first ran the same multiple regression analysis with a larger sample in Study 2. As shown in Table 7, our model showed a similar pattern to Study 1, indicating a significant effect of the regression model, *F*(4, 295) = 35.52, *p* < .001, accounting 22% of the variances of SNS addiction, while showing the highest contribution of social comparison of ability (*β* = .28, *p <* .001), followed by the perceived relative deprivation of material wealth (*β* = .12, *p* = .014), and perceived relative deprivation of social support (*β* = .13, *p* = .009). For a comparison reason, we ran the same analysis for Study 1 using a 3-item SPRDS to measure perceived relative deprivation of social support with reverse items excluded from the scale.

**Table 7:** Comparison of the identical regression models using samples in Study 1 and Study 2.

		SNS addiction			
	Predictors	*b* (*se*)	95% CI for *b*	*β*	*sr* ^2^
Study 1 (n = 103)	SC-ability	.38 (0.11)	.17, .60	.42***	.31
	SC-opinion	.01 (0.12)	-.23, .25	.01	.006
	RD Social Support	.02 (0.07)	-13, .16	.02	.02
	RD Material Wealth	.12 (0.08)	-.03, .27	.16	.14
Study 2 (n = 500)	SC-ability	.22 (0.04)	.14, .30	.28***	.21
	SC-opinion	.06 (0.04)	-.02, .13	.07	.06
	RD Social Support	.08 (0.03)	.02, .14	.13**	.10
	RD Material Wealth	.07 (0.03)	.02, .13	.12*	.08

*Note*. SC = Social Comparison; RD = Relative Deprivation. SNS = Social Network Site. *sr*^2^ = semi-partial correlation squared.

* p < .*05*.

** p < .*01*

*** *p* < .001.

**Fig 2:**
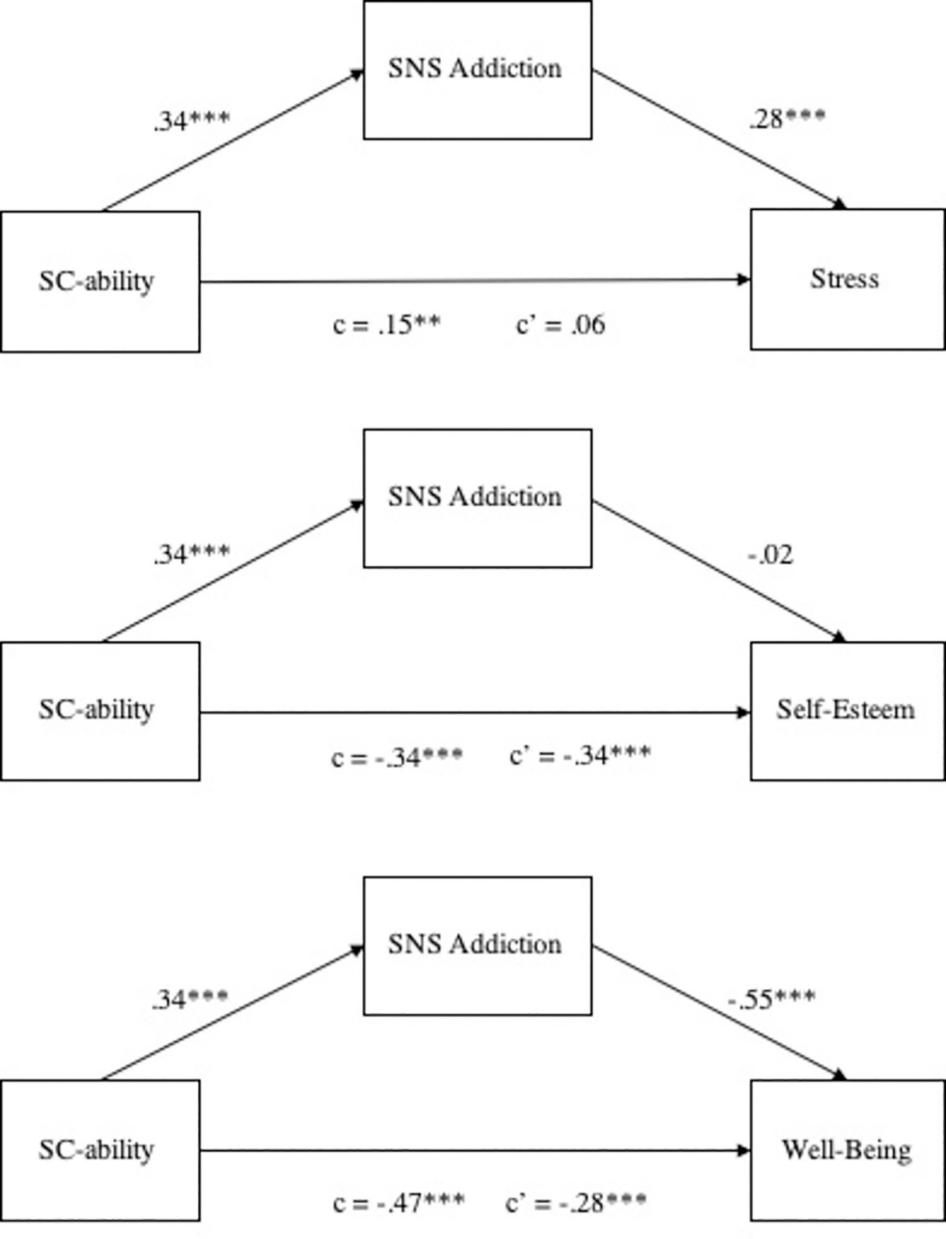
The mediating role of SNS addiction on the relations between social comparison of ability and stress, self-esteem and well-being.

Subsequently, we ran three separate mediation analyses to test our hypotheses H3a, H3b, and H3c, a mediating role of SNS addiction in relations between social comparison of ability and stress, self-esteem, and well-being (see Fig 2). Bootstrapped mediation analyses with 10,000 samples revealed that SNS addiction mediated the relation of social comparison of ability and stress (95% BCa CI of .05 and .15; total effect = .15; indirect effect = .10, *SE* = .03), indicating a full mediation, replicating Study 1. The second mediation analysis revealed that SNS addiction did not mediate the relation between social comparison of ability and self-esteem (95% BCa CI of -.06 and .04; total effect = -.34; indirect effect = -.01, *SE* = .03), replicating the non-significant result observed in Study 1. Lastly, SNS addiction mediated the relations between social comparison of ability and well-being (95% BCa CI of -.26 and -.11; total effect = -.47; indirect effect = -.18, *SE* = .04) indicating a partial mediation.

**Fig 3:**
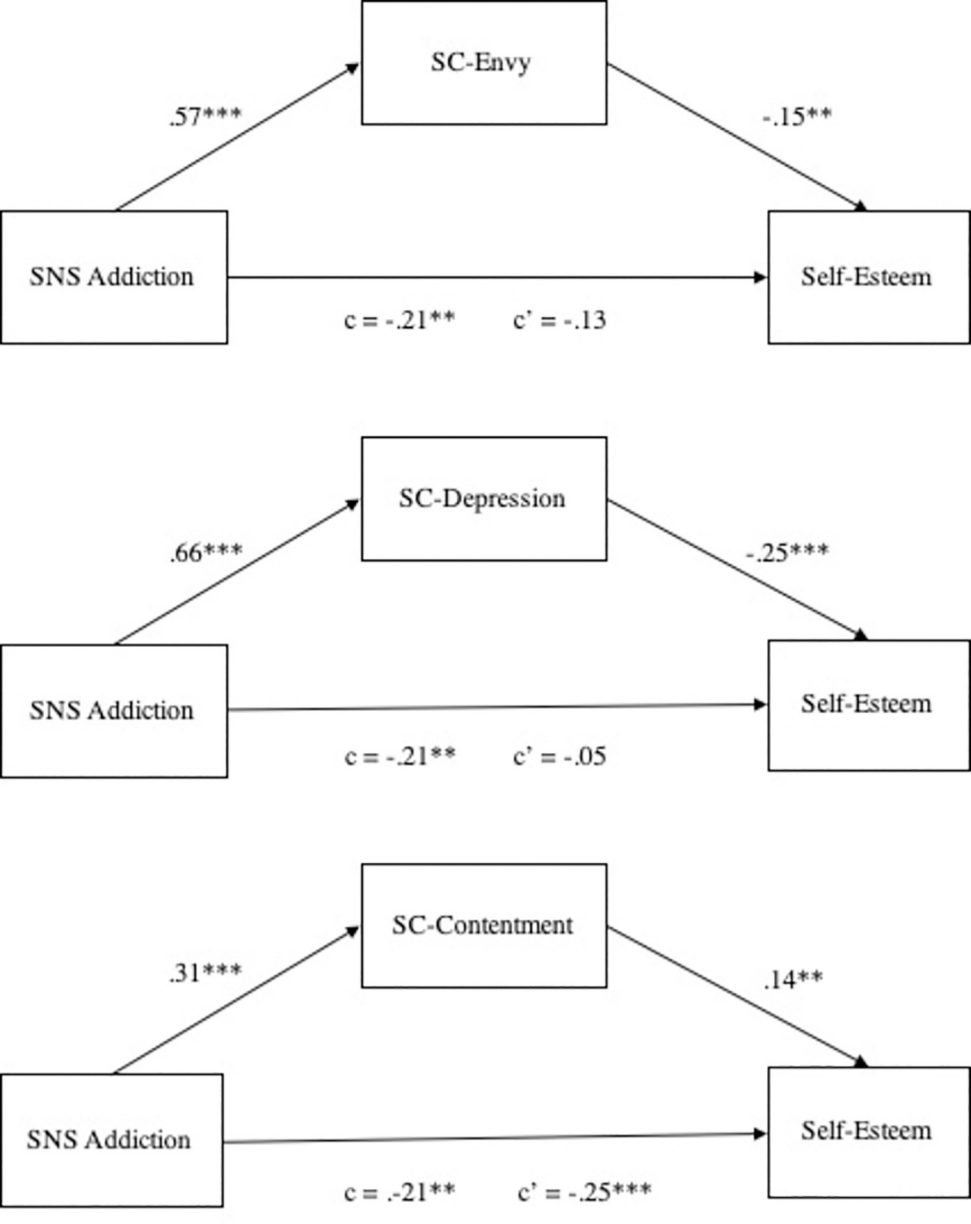
The mediating role of SC-envy and SC-depression on the relation between SNS addiction and self-esteem. Note. In the mediation model via SC-contentment, due to the indirect effect (.04) being positive and the direct effect being negative (-.25), the indirect and direct effects cancelled each other out (c = -.21; inconsistent mediation; [85]). In this case, an appropriate interpretation of the size of the total effect can be the combination of the absolute values of indirect and direct effects (c = .29; [86]).

Next, we ran three separate mediation analyses to test our Hypothesis 4a assessing the mediating role of the feelings of contrastive emotions (i.e., envy, anger, and depression) on the relation between SNS addiction and self-esteem (see Fig 3). Bootstrapped mediation analyses revealed that SC-envy and SC-depression fully mediated the relation between SNS addiction and self-esteem (SC-envy: 95% BCa CI of -.16 and -.02; total effect = -.21; indirect effect = -.13, *SE* = .07; SC-depression: 95% BCa CI of -.25 and -.08; total effect = -.21; indirect effect = -.16, *SE* = .05; see Fig 2) but not through SC-anger (95% BCa CI of -.06 and .002; total effect = -.21; indirect effect = -.03), indicating that the association between SNS addiction and self-esteem can be explained via the feelings of envy and depression after engaging in SNS social comparisons.

To test our Hypothesis 4b, we ran two separate mediation analyses, assessing the mediating role of the positive emotions (i.e., inspiration and contentment) on the relation between SNS addiction and self-esteem. Bootstrapped mediation analyses revealed that this relation was not mediated by SC-inspiration (95% BCa CI of -.07 and .04; total effect = -.22; indirect effect = -.01, *SE* = .03) but partially mediated by SC-contentment (95% BCa CI of .01 and .08; total effect = -.21[.29]; indirect effect = .04, *SE* = .02), indicating that the association between SNS addiction and higher self-esteem can partially be explained via the feeling of contentment after engaging in SNS social comparisons (see Fig 3). Overall, our Hypotheses 4a and 4b were partially confirmed. All regression and mediation analyses conducted in Study 2 showed similar results after controlling for age and gender.

**Fig 4:**
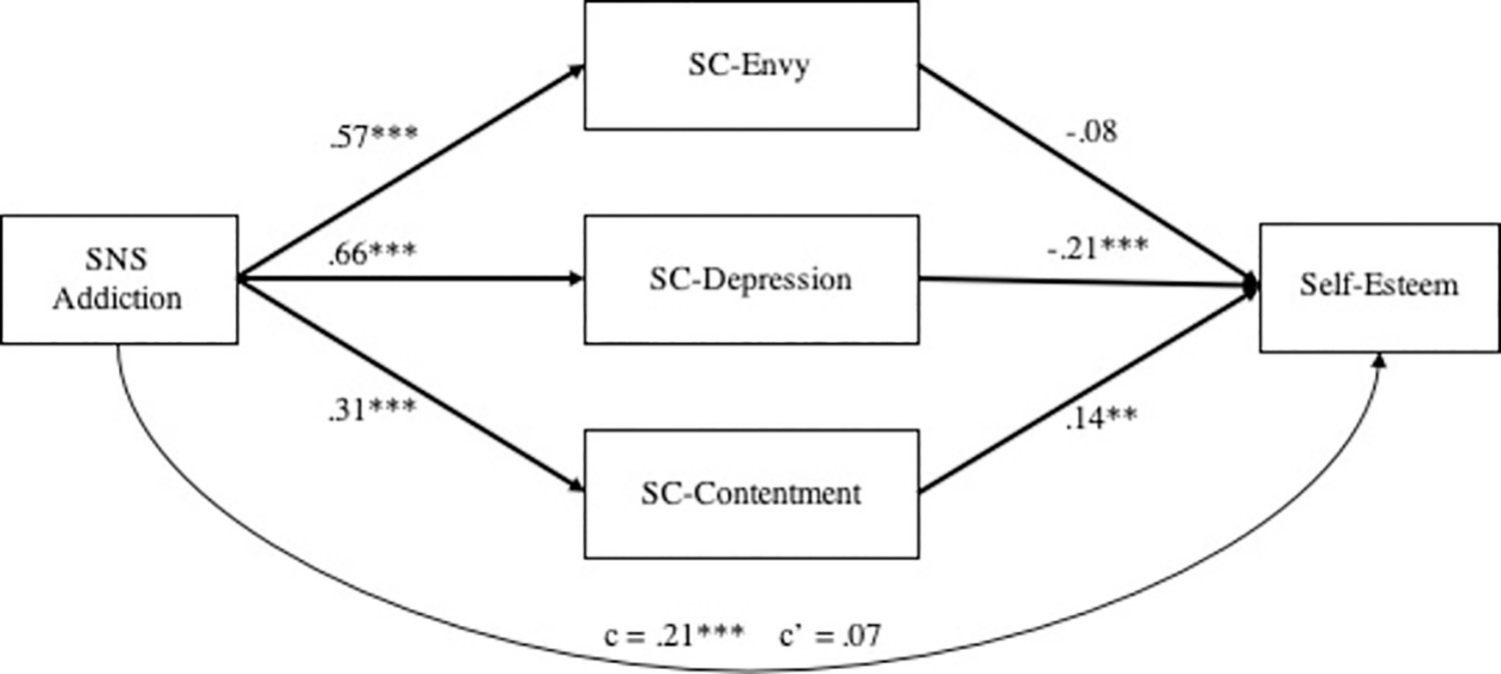
The jointly mediating role of SC-envy, SC-depression, and SC-contentment between SNS addiction and self-esteem.

Lastly, for an exploratory purpose, we tested a multi-mediation model including three mediators that significantly contributed to explaining the relation between SNS addiction and self-esteem (SC-envy, SC-depression, and SC-contentment) in one model. Fig 4 depicted the relations among social comparison elicited emotions of envy, depression and contentment and their jointly mediating role of the effect of SNS addiction on self-esteem. Bootstrapped mediation analyses revealed that the multi-mediation model was significant (95% BCa CI of -.34 and -.09; total effect = -.21; indirect effect = -.14, *SE* = .05), indicating a full mediation (direct effect = -.07, CIs = -.22, .07). SC-Depression (indirect effect = -.14; CIs = -.23, -.05) and SC-Contentment (indirect effect = .04; CIs = .01, .08) significantly mediated the relation whereas SC-Envy did not (indirect effect = -.05; CIs = -.12, .02). This joint model, however, must give caution to interpretations because our mediators were correlated with each other. Future researchers should test more precise hypotheses to build a parsimonious model.

### Discussion

Using a sample that matched the gender distribution, we largely replicated the findings observed in Study 1. Our results demonstrated the significant contribution of social comparison relevant constructs to SNS addiction. In Study 2, we showed that the ability comparison tendency was indeed the strongest predictor on SNS addiction. As hypothesized, SNS addiction mediated the relations between social comparison of ability and well-being and stress indicating that people with a higher tendency to compare themselves with others on social outcomes are more likely to experience decreased well-being and increased stress via SNS addiction. Our findings provide robust evidence supporting the crucial role of the tendency to engage in social comparisons of ability to understanding SNS addiction. Identical to Study 1, the relation between the ability comparison tendency and self-esteem was not mediated by SNS addiction. To further investigate this relation, we tested the mediating role of SNS social comparison emotions on the relation between SNS addiction and self-esteem and found that two contrastive upward social comparison emotions (envy and depression but not anger) fully mediated the relation between SNS addiction and self-esteem. This finding stresses the role of social comparison elicited negative emotions playing in the link between problematic SNS use and lower self-esteem. On the flip side, our mediation analyses also showed that the feeling of contentment after making SNS social comparisons also mediated the link between problematic SNS use and higher self-esteem, supporting our hypothesis that a positive experience after engaging in SNS social comparisons might boost self-esteem, even if not strong enough to surpass the effects of the negative experiences. The role of positive social comparison emotions during SNS use might potentially explain why individuals continue to use SNS despite the negative consequences.

Contrary to Study 1, we observed significant, although relatively small, contribution of personal relative deprivations of material wealth and social support to explaining the variances of SNS addiction (Table 7). It is possible that the bigger sample size in Study 2 compared to the sample size in Study 1 allowed the detection of this effect. However, it has to be considered that the two samples in Study 1 and Study 2 also varied in other characteristics (e.g., age, gender balance, education). Using a more representative and larger sample, Study 2 allows more robust conclusions.

## General discussion

Previous research has shown that excessive use of SNSs can lead to negative outcomes for individuals. However, the existing literatures did not provide a clear picture how social comparison related antecedents and consequences of SNS use contribute to well-being, stress, and self-esteem when SNS use becomes excessive. Hence, we investigated the influence of social comparison on SNS addiction and the experience of social comparison-related emotions as an outcome of SNS use across two studies. Further, we tested whether SNS addiction mediates the effects of the ability comparison tendency on stress, self-esteem, and well-being. In Study 1, as hypothesized, the tendency to engage in social comparisons of ability (but not of opinion; H1) predicted SNS addiction over and above the perceived relative deprivation of wealth, and the perceived relative deprivation of social support (H2). Moreover, SNS addiction fully mediated the relation of the effect of social comparison of ability on stress (H3a) but not the effect on self-esteem (H3b). In Study 2, we replicated the results observed in Study 1 and showed that SNS addiction also mediated the relation between the social comparison tendency of ability and well-being (H3c). Finally, envy and depression (but not anger) felt after engaging in SNS social comparisons fully mediated the relation between SNS addiction and lower self-esteem (H4a) whereas contentment (but not inspiration) felt after engaging in SNS social comparisons partially mediated the relation between SNS addiction and higher self-esteem (H4b).

Our finding also suggests that not all negative consequences of social comparisons can predict uncontrollable SNS behaviors. We showed that the perception of relative deprivation of material wealth and social support were not associated with our measure of SNS addiction using a convenient sample in Study 1. This result was rather surprising given that the tendency to engage in ability-based social comparisons is often strongly associated with feeling deprived in material wealth [62, 87]. However, using a larger and broader sample according to demographic characteristics in Study 2, we could detect small to moderate effects (*β*s = .12, .13) of the predictive power, although the predictive power of the ability comparison tendency was the strongest (*β* = .28). This pattern might imply that the perception of relative deprivation and social comparison tendencies might be dissociable psychological constructs for explaining SNS behaviors. For example, those who often feel relatively deprived might utilize SNSs for means to gain deprived resources but once gained, the feeling of relative deprivation would be diminished and thus SNS activity would no longer serve its purpose. In contrast, engaging in social comparisons of ability directly affects the reward system [88] whereby constant comparisons, thus excessive SNS use, might increase expectations for chances of feeling good about oneself compared to others (i.e., feeling of contentment).

Whilst abundant research has mainly focused on the association between detrimental effects and excessive SNS use [40], our findings point to both sides of the coin. The significant mediating roles of both negative and positive social comparison emotions from SNS addiction to self-esteem might help disentangle the complex dynamic of the behavioral addiction on SNS. SNS users who report excessive use might suffer from detrimental psychological effects, partly, as a result of making adverse social comparisons readily available on SNS platforms. Yet, our findings imply that the social values gained (i.e., information to engage in self-evaluation) by engaging in social comparisons of ability and the emotional reward by making contrastive downward comparisons might also be attributable to the repeated use of SNSs.

The link between SNS addiction and self-esteem explored in the current study is in line with previous studies showing an overall negative association between problematic SNS use and low self-esteem [54]. Our findings extend the previous research by uncovering the mechanism through the lens of social comparison emotions. The results showed that those who reported higher level of SNS addiction also reported lower self-esteem and this relation was fully mediated by the feelings of envy and depression (on separate mediations) after engaging in SNS social comparisons. As social comparison theories predict, easy adverse social comparison opportunities available on SNS platforms can be toxic to keep one’s positive self-view, especially when the comparison targets are most likely to be portrayed in an idealized way [34]. Equally harmful might be for those who report low self-esteem constantly going back to engage in SNS activities in a hope for boosting self-esteem because individuals with low self-esteem tend to perceive upward comparisons as more negative than those with high self-esteem [45]. Nevertheless, our data also showed that people can still boost their self-esteem when engaging in SNS activities via the feeling of contentment after making SNS social comparisons. Our findings imply that one of the reasons why people repeatedly fall back to using SNS despite the overall negative effects on well-being, stress, and self-esteem might be the positive feelings acquired by engaging in contrastive downward comparisons. Contrarily, the feeling of inspiration after making upward comparisons did not significantly mediate the relation between SNS addiction and higher self-esteem. This result raises a question whether SNS induced inspiration might be short-lived or not as effective as in real-life experience of inspiration by others. Future research can address specific mechanisms each social comparison emotion might play on SNS activities for maintaining or failing to maintain a positive self-view.

Our findings are also in line with previous research showing that making ability-based social comparisons among SNS users can lead to decreased subjective well-being [83]. In our study, the associations between the tendency to make social comparisons of ability and decreased well-being and increased stress were through SNS addiction. This finding again emphasizes the important role social comparison plays in understanding problematic SNS use, which might lead to detrimental effects on mental health. As most SNS providers often claim, the intended functions of SNS are mainly to have easier ways to keep up with friends and to stay in contact with important social groups. And SNSs are indeed shown to enhance group cohesion [5]. However, our findings suggest that once SNS platforms are used as means to make easily available social comparisons of ability, SNS activities might have detrimental consequences. Future longitudinal studies are highly recommended to precisely assess the directionality of the effects. For example, on the one hand, the tendency to engage in social comparisons might exacerbate SNS addiction but on the other hand, SNS addiction might boost engagement of making SNS social comparisons. This potential loop can be verified in more controlled experiments or longitudinal studies. Further research investigating ways to promote healthy user behavior in SNSs is also highly warranted.

Another shortfall of the current research is that the samples used in both studies were not clinical populations. Investigating the behavior of SNS users showing a high SNS addiction tendency might verify a fuller mechanism between social comparison tendency and SNS addiction. Whether individuals who tend to lose control over their behavior might show an identical mechanism seen in our finding is an open question. Future researchers are encouraged to examine the boundaries for clinical and non-clinical SNS behaviors and social comparison elicited emotions with more precise hypotheses that can test parsimonious models.

Lastly, the present studies were cross-sectional, leaving open the possibility that the predictors we observed do not suggest a causal relationship with SNS addiction. Longitudinal studies are thus needed to examine intra-individual changes in the social comparison tendency and its connected emotions in relation to excessive SNS use. Yet, our results provide some ground work to future researchers exploring the social comparison constructs in uncontrollable SNS use.

## Supporting information

S1 Table German version of Personal Relative Deprivation Scale (PRDS). (DOC)

S2 Table Summary of exploratory factor analyses for the original five-item German-translated PRDS. (DOCX)

S3 Table Summary of exploratory factor analyses for the 3-item German-translated PRDS. (DOCX)

S4 Table German version of Social Personal Relative Deprivation Scale (SPRDS). (DOC)

S5 Table Summary of exploratory factor analyses for the five-item German-translated SPRDS. (DOC)

S6 Table Summary of exploratory factor analyses for the 3-item German-translated SPRDS. (DOC)

S7 Table German version of Bergen Social Media Addiction Scale (BSMAS). (DOC)

S1 Scale German version of self-esteem measure. (DOC)

S2 Scale German version of perceived stress measure. (DOC)
