# Supplementary material for: The contributions of social comparison to social network site addiction
Source: PLoS One. 2021 Oct 28;16(10):e0257795. doi: 10.1371/journal.pone.0257795 (PMC8553147; doi:10.1371/journal.pone.0257795)
Supplement: S1 Table — (DOC) [file pone.0257795.s001.doc]

**S1 Table. German Version of Personal Relative Deprivation Scale (PRDS).**

| **Items** |
| --- |
| 1. Ich fühle mich benachteiligt, wenn ich darüber nachdenke, was ich habe, verglichen mit dem, was andere Personen wie ich haben.  [I feel deprived when I think about what I have compared to what other people like  me have.] |
| 2. Ich fühle mich privilegiert im Vergleich zu anderen Personen wie mir.  [I feel privileged compared to other people like me.] |
| 3. Ich fühle mich verärgert, wenn ich sehe, wie wohlhabend andere Personen wie ich zu sein scheinen.  [I feel resentful when I see how prosperous other people like me seem to be.] |
| 4. Wenn ich das, was ich habe, mit dem vergleiche, was andere Leute wie ich haben, wird mir klar, dass es mir ziemlich gut geht.  [When I compare what I have with what others like me have, I realize that I am quite  well off.] |
| 5. Ich fühle mich unzufrieden mit dem, was ich habe, verglichen mit dem, was andere Personen wie ich haben.  [I feel dissatisfied with what I have compared to what other people like me have] |

^a^ Items 2 and 4 were reverse-coded.

^b^ Participants were given a 6 point scale; 1 = *Ich stimme überhaupt nicht zu* [*strongly disagree*], 2 = *Ich stimme nicht zu* [*disagree*], 3 = *Ich stimme eher nicht zu* [*somewhat disagree*], 4 = *Ich stimme eher zu* [*somewhat agree*], 5 = *Ich stimme zu* [*agree*], 6 = *Ich stimme voll und ganz zu* [*strongly agree*].

^c^ Original items are presented in brackets.
