# Supplementary material for: The contributions of social comparison to social network site addiction
Source: PLoS One. 2021 Oct 28;16(10):e0257795. doi: 10.1371/journal.pone.0257795 (PMC8553147; doi:10.1371/journal.pone.0257795)
Supplement: S2 Table — (DOCX) [file pone.0257795.s002.docx]

**S2 Table.** **Summary of Exploratory Factor Analyses for the Original Five-Item German-Translated PRDS.**

|  | Study 1 (*N* = 103) | | | Study 2 (N = 500) | | |  |
| --- | --- | --- | --- | --- | --- | --- | --- |
|  | Communalities and Rotated Factor Loadings | | | | | |  |
| *Scale Items* | Communality | Factor 1 | Factor 2 | Communality | Factor 1 | Factor 2 | |
| 1. I feel deprived when I think about what I have compared to what other people like me have. | .80 | .86 | -.22 | .79 | .87 | -.18 | |
| 2. I feel privileged compared to other people like me. | .82 | .43 | .80 | .71 | .22 | .87 | |
| 3. I feel resentful when I see how prosperous other people like me seem to be. | .72 | .79 | -.31 | .78 | .83 | -.31 | |
| 4. When I compare what I have with what others like me have, I realize that I am quite well off. | .76 | .65 | .58 | .70 | .59 | .59 | |
| 5. I feel dissatisfied with what I have compared to what other people like me have. | .71 | .75 | -.39 | .71 | .83 | -.16 | |
| Eigenvalues |  | 2.54 | 1.27 |  | 2.53 | 1.27 | |
| % of variance |  | 50.73 | 25.34 |  | 50.64 | 25.38 | |

*^a^*Items 2 and 4 were reverse-coded.
