# Supplementary material for: The contributions of social comparison to social network site addiction
Source: PLoS One. 2021 Oct 28;16(10):e0257795. doi: 10.1371/journal.pone.0257795 (PMC8553147; doi:10.1371/journal.pone.0257795)
Supplement: S3 Table — (DOCX) [file pone.0257795.s003.docx]

**S3 Table.** **Summary of Exploratory Factor Analyses for the 3-Item German-Translated PRDS.**

|  | Study 1 (*N* = 103) | | Study 2 (N = 500) | |
| --- | --- | --- | --- | --- |
|  | Communalities and Rotated Factor Loadings | | | |
| *Scale Items* | Communality | Factor 1 | Communality | Factor 1 |
| 1. I feel deprived when I think about what I have compared to what other people like me have. | .80 | .89 | .80 | .90 |
| 2. I feel resentful when I see how prosperous other people like me seem to be. | .72 | .85 | .76 | .87 |
| 3. I feel dissatisfied with what I have compared to what other people like me have. | .69 | .83 | .73 | .85 |
| Eigenvalues |  | 2.21 |  | 2.30 |
| % of variance |  | 73.60 |  | 76.55 |
