# Supplementary material for: The contributions of social comparison to social network site addiction
Source: PLoS One. 2021 Oct 28;16(10):e0257795. doi: 10.1371/journal.pone.0257795 (PMC8553147; doi:10.1371/journal.pone.0257795)
Supplement: S4 Table — (DOC) [file pone.0257795.s004.doc]

**S4 Table. German Version of Social Personal Relative Deprivation Scale (SPRDS).**

| **Items** |
| --- |
| 1. Wenn ich darüber nachdenke, wie viele enge Freundschaften ich im Vergleich mit anderen Leuten wie mir habe, fühle ich mich benachteiligt.  [​I feel deprived when I think about how many close relationships I have compared to  what other people like me have] |
| 2. Wenn ich an meine engen Freundschaften denke, fühle ich mich im Vergleich mit anderen Personen wie mir privilegiert.  [Thinking of my close relationships, I feel privileged compared to other people like  me] |
| 3. Ich fühle mich verärgert, wenn ich sehe, wie viele enge Freundschaften andere Personen wie ich zu haben scheinen.  [I feel resentful when I see how many close​ relationships other people like me seem to  have] |
| 4. Wenn ich die engen Freundschaften, die ich habe, mit denen vergleiche, die andere Personen wie ich haben, wird mir klar, dass es mir ziemlich gut geht.  [When I compare the close relationships I have with the​ ​close relationships  others like me have, I realize that I am quite well] |
| 5. Ich fühle mich unzufrieden mit den engen Freundschaften, die ich habe, verglichen mit denen, die andere Personen wie ich haben.  [I feel dissatisfied with the close relationships I have compared to those that other people  like me have] |

^a^ Items 2 and 4 were reverse-coded.

^b^ Participants were given a 6 point scale; 1 = *Ich stimme überhaupt nicht zu* [*strongly disagree*], 2 = *Ich stimme nicht zu* [*disagree*], 3 = *Ich stimme eher nicht zu* [*somewhat disagree*], 4 = *Ich stimme eher zu* [*somewhat agree*], 5 = *Ich stimme zu* [*agree*], 6 = *Ich stimme voll und ganz zu* [*strongly agree*].

^c^ Original items are presented in brackets.
