# Supplementary material for: The contributions of social comparison to social network site addiction
Source: PLoS One. 2021 Oct 28;16(10):e0257795. doi: 10.1371/journal.pone.0257795 (PMC8553147; doi:10.1371/journal.pone.0257795)
Supplement: S5 Table — (DOC) [file pone.0257795.s005.doc]

**S5 Table.** Summary of Exploratory Factor Analyses for the Five-Item German-Translated SPRDS.

|  | Study 1 (*N* = 103) | | Study 2 (*N* = 500) | | |
| --- | --- | --- | --- | --- | --- |
|  | Communalities and Rotated Factor Loadings | | | | |
| *Scale Items* | Communality | Factor 1 | Communality | Factor 1 | Factor 2 |
| 1. I feel deprived when I think about  how many close relationships I have compared to what other people like me have. | .71 | .84 | .75 | .82 | -.28 |
| 2. Thinking of my close relationships, I feel privileged compared to other people like me. | .31 | .55 | .84 | .57 | .72 |
| 3. I feel resentful when I see how many close​ relationships other people like me seem to have. | .51 | .72 | .78 | .79 | -.39 |
| 4. When I compare the close relationships I have with the​ ​close relationships others like me have, I realize that I am quite well. | .56 | .75 | 80 | .71 | .54 |
| 5. I feel dissatisfied with the close relationships I have compared to those that other people like me have. | .67 | .82 | .65 | .72 | -.36 |
| Eigenvalues |  | 2.75 |  | 2.66 | 1.17 |
| % of variance |  | 55.01 |  | 53.22 | 23.31 |

*^a^*Items 2 and 4 were reverse-coded.
