# Supplementary material for: The contributions of social comparison to social network site addiction
Source: PLoS One. 2021 Oct 28;16(10):e0257795. doi: 10.1371/journal.pone.0257795 (PMC8553147; doi:10.1371/journal.pone.0257795)
Supplement: S6 Table — (DOC) [file pone.0257795.s006.doc]

**S6 Table.** Summary of Exploratory Factor Analyses for the 3-Item German-Translated SPRDS.

|  | Study 1 (*N* = 103) | | Study 2 (*N* = 500) | |
| --- | --- | --- | --- | --- |
|  | Communalities and Rotated Factor Loadings | | | |
| *Scale Items* | Communality | Factor 1 | Communality | Factor 1 |
| 1. I feel deprived when I think about  how many close relationships I have compared to what other people like me have. | .81 | .90 | .75 | .87 |
| 2. I feel resentful when I see how many close​ relationships other people like me seem to have. | .66 | .82 | .78 | .88 |
| 3. I feel dissatisfied with the close relationships I have compared to those that other people like me have. | .69 | .83 | .65 | .81 |
| Eigenvalues |  | 2.16 |  | 2.18 |
| % of variance |  | 72.09 |  | 72.67 |
