# Supplementary material for: The contributions of social comparison to social network site addiction
Source: PLoS One. 2021 Oct 28;16(10):e0257795. doi: 10.1371/journal.pone.0257795 (PMC8553147; doi:10.1371/journal.pone.0257795)
Supplement: S7 Table — (DOC) [file pone.0257795.s007.doc]

**S7 Table. German Version of Bergen Social Media Addiction Scale (BSMAS).**

Wie oft haben Sie im letzten Jahr…

[How often during the last year have you …]

| **Items** |
| --- |
| 1. …viel Zeit mit dem Nachdenken über Social Media oder geplante Nutzung von Social Media verbracht?  [​…spent time thinking about social media or the usage of social media?] |
| 2. … den Drang verspürt, Social Media immer mehr zu nutzen?  [​…felt an urge to use social media more and more?] |
| 3. …Social Media benutzt, um persönliche Probleme zu vergessen?  […used social media to forget about personal problems?] |
| 4. …versucht, die Benutzung von Social Media ohne Erfolg einzuschränken?  […tried to cut down on the use of social media without success?] |
| 5. … Social Media so viel genutzt, dass es einen negativen Einfluss auf Ihren Job/ Ihr Studium hatte?  […used social media so much that it has had a negative impact on your  job/studies?] |
| 6. … haben Sie sich unruhig oder bedrückt gefühlt, wenn Ihnen Social Media nicht zur Verfügung stand?  [become restless or troubled if you have been prohibited from using social media?] |

^a^ Participants were given a 5 point scale; 1 = *Nie* [*never*], 5 = *Sehr oft* [*very often*].

^b^ Original items are presented in brackets.
