# Supplementary material for: The contributions of social comparison to social network site addiction
Source: PLoS One. 2021 Oct 28;16(10):e0257795. doi: 10.1371/journal.pone.0257795 (PMC8553147; doi:10.1371/journal.pone.0257795)
Supplement: S1 Scale — (DOC) [file pone.0257795.s008.doc]

**S1 Scale. German Version of Self-Esteem Measure**

Ich habe ein hohes Selbstwertgefühl.

[I have a high self-esteem]

1. *Trifft überhaupt nicht auf mich zu*. [*not very true of me*]

2.

3.

4.

5. *Trifft vollkommen auf mich zu*. [*very true of me*]
