# Supplementary material for: The contributions of social comparison to social network site addiction
Source: PLoS One. 2021 Oct 28;16(10):e0257795. doi: 10.1371/journal.pone.0257795 (PMC8553147; doi:10.1371/journal.pone.0257795)
Supplement: S2 Scale — (DOC) [file pone.0257795.s009.doc]

**S2 Scale. German Version of Perceived Stress Measure**

Wie viel Stress (z.B.:, aufgrund von Ärger, Beanspruchungen) hatten Sie in letzter Zeit?

[How much stress (e.g., because of hassles, demands) were you under recently?]

1. *Sehr wenig* [*felt very slightly or not at all*]

2.

3.

4.

5. *Sehr viel* [*felt very much*]
